# Using 3D models in orthopedic oncology: presenting personalized advantages in surgical planning and intraoperative outcomes

**DOI:** 10.1186/s41205-018-0035-6

**Published:** 2018-11-26

**Authors:** Thipachart Punyaratabandhu, Peter C. Liacouras, Sutipat Pairojboriboon

**Affiliations:** 1 Department of Orthopaedics, Phramongkutklao Hospital and College of Medicine, Bangkok, Thailand; 2 3D Medical Application Center, Department of Radiology, Walter Reed National Military Medical Center, Bethesda, USA; 3 Radiology and Radiological Services & Naval Postgraduate Dental School, Uniform Services University of the Health Sciences, Bethesda, USA

**Keywords:** 3D printing, Bone tumors model, Acetabular deficiency, CT + MRI images fusion, Anatomical model

## Abstract

**Background:**

Three Dimensional (3D) printed models can aid in effective pre-operative planning by defining the geometry of tumor mass, bone loss, and nearby vessels to help determine the most accurate osteotomy site and the most appropriate prosthesis, especially in the case of complex acetabular deficiency, resulting in decreased operative time and decreased blood loss.

**Methods:**

Four complicated cases were selected, reconstructed and printed. These 4 cases were divided in 3 groups of 3D printed models. Group 1 consisted of anatomical models with major vascular considerations during surgery. Group 2 consisted of an anatomical model showing a bone defect, which was intended to be used for substantial instrumentation, pre-operatively. Group 3 consisted of an extra-compartmental bone tumor which displayed a deteriorated cortical outline; thus, using CT and MRI fused images to reconstruct the model accurately. An orthopedic surgeon created the 3D models of groups 1 and 2 using standard segmentation techniques. Because group 3 required complex techniques, an engineer assisted during digital model construction.

**Results:**

These models helped to guide the orthopedic surgeon in creating a personalized pre-operative plan and a physical simulation. The models proved to be beneficial and assisted with all 4 cases, by decreasing blood loss, operative time and surgical incision length, and helped to select the appropriate acetabular supporting ring in complex acetabular deficiency, pre-operatively.

**Conclusion:**

Qualitatively, using 3D printing in tumor cases, provides personalized advantages regarding the various characteristics of each skeletal tumor.

## Background

Bone tumors are generally rare diseases. They can occur in various locations of the skeletal system which sometimes are difficult to access such as the peri-acetabular area [1] and the posterior part of the distal femur and proximal tibia and near the neurovascular bundles [2]. Moreover, some conditions such as bone metastasis from the thyroid or renal cancer can cause high volume bleeding conditions [3]. A thorough pre-operative plan to reduce operative time is critical. Currently, 3D (3 dimensional) printing technology has gained popularity in medicine especially in orthopedic oncology because of the ability to create patient-specific, accurate, and high fidelity models that can be used to create patient-specific operational plans and prostheses. Much literature supports using 3D printing in clinical practice with most revolving around using the 3D model as a physical simulation to aid in pre-operative planning and the fabrication process of customized 3D printed implants. Examples of clinical use in the 3D model are preshaping plates for minimally invasive fixation in calcaneal fracture [4], pre-operative planning for minimally invasive fixation in comminuted fracture of tibial plateau [5] and a combination of 3D printing and computer-assisted virtual surgery regarding the acetabular fracture [6]. In addition, some bone tumor cases have a soft tissue extension from the cortex; thus, mandating the use of more advanced reconstruction techniques combining computed tomography (CT) and magnetic resonance imaging (MRI) creating the subject-specific 3D model were performed [7]. Segmentations which use such fused imaging offers more accurate information than either CT or MRI alone by combining the different tissue sensitivities of each modality. While CT images are excellent for segmenting bony structures, MRI images play a major role to segment non-bony structures [7]. Customized 3D printed implants are increasingly popular and are being currently manufactured for a variety of anatomic sites. These personalized advantages of patient-matched and unique skeletal structures are featured in studies including 3D printed metacarpal prosthesis [8], customized 3D printed knee prosthesis [9] and customized complicated acetabular prosthesis [10].

Essential to the successful integration of this technology is the inter-professional cooperation and communication between the orthopedic surgeon and the engineer as they have technological and educational differences. For example, the engineer may not be familiar with anatomy, epidemiology, surgical approach and resection/reconstruction procedures. In contrast, the orthopedic surgeon may not know the numerous steps of the fabrication process of a 3D-printed physical model that can be described as a combination of 3D volumetric imaging acquisition, image postprocessing, and 3D printing technology. Postprocessing of Digital Imaging and Communications in Medicine (DICOM) imaging data is an important step to generate the Standard Tessellation Language (STL) file that is able to be read by 3D printers. Computer-aided design (CAD) software is for refining or instrument/device designing, model quality checking, and file fixing. 3D printers use data encoded in STL file format to successively fuse thin layers of material to create a 3D object [11]. The current standards classifications comprise seven specific groups of 3D printing technologies. These are vat photopolymerization, material jetting, binder jetting, material extrusion, powder bed fusion, sheet lamination and directed energy deposition [12–14]. Each technology has strengths and weaknesses as it pertains to its uses in clinical 3D printing. When the orthopedic surgeon is unaware of the limitations or resources of the engineer, and vice versa, developing modified anatomical model, custom cutting guide, and patient-matched prosthesis would possibly become time-consuming and errors.

The objective of this study was to present in vivo surgical outcomes of the procedures using 3D printed models as a 3D physical surgical simulation and resources in pre-operative planning. For most of the models in this report, the orthopedic surgeon, who performed the surgery, performed image processing from DICOM to STL file and the resulting 3D printed anatomical model. However, some complicated situations occurred. For example, to create a 3D model of the chondrosarcoma with cortical breakthrough and soft tissue extension, we cooperated with an engineer to use more advanced modeling techniques such as fusing CT and MRI images and STL registration.

## Methods

The cases were divided in three groups based on their intended use, which may also roughly correlate to how the US Food and Drug Administration (FDA) views these applications [15]. Group I comprised anatomical models. Group II consisted of modified anatomical models while Group III comprised virtual surgical planning with templates. However, in this study, we focused only on group I. Anatomical models were defined as models representing the scanned anatomy intended for visualization, surgical planning, education, informed consent and reference during surgery. The intended use involved a 3D reference of the anatomy to aid surgical planning.

The institutional review board approved the present study at the Royal Thai Army Medical Department of Phramongkutklao Hospital. Consent to participate in this research was obtained from all patients. Four cases were selected to be printed as group I.

Group 1 comprised anatomical models with major vascular consideration during surgery. Group 2 comprised anatomical models with bone defects, which were intended to be used for substantial instrumentation, pre-operatively. Group 3 comprised bone tumor models with soft tissue extension, requiring MRI image acquisition, which were intended to be used for tumor margin evaluation. Image acquisition of groups 1 and 2 was derived only from CT scans. The image postprocessing and printing process were created by an orthopedic surgeon, who became familiar with material extrusion or fused deposition modeling (FDM) 3D printers. In addition, the software converted DICOM data to STL files using the MIMICS Innovation Suite, Version 19.0 (MIMICS – Materialise Interactive Medical Image Control System Software, Materialise, Leuven, Belgium) at the 3D Medical Application Center, Walter Reed National Military Medical Center, Bethesda, USA. Group 3 generating models with assistance from an engineer due to the need to use advanced techniques like CT + MRI fused images. Mimics Medical (Materialise) software was used to perform the registration. The registration can be done by picking corresponding points on the 2 images sets. However when we have scans that cross an articulating joint, sometimes the STL files representing the bones of that joint need to be registered to the MRI individually.

**Fig. 1:**
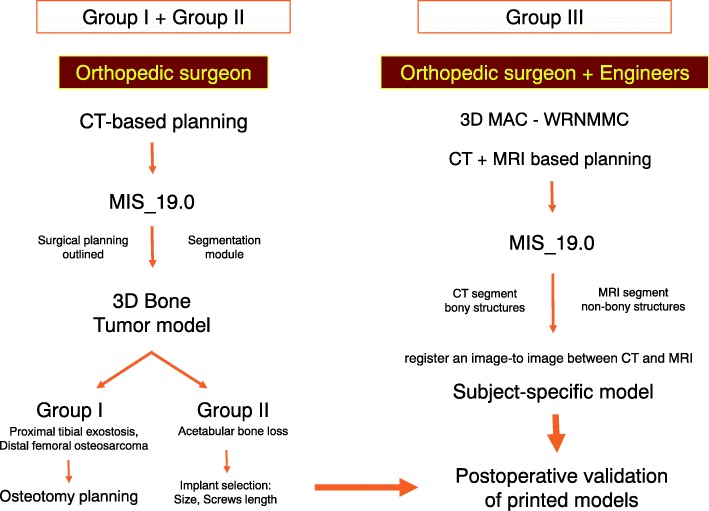
Workflow for creating and using 3D printed presurgical planning models for bone tumors surgeries. This scheme shows the effectiveness of achieving personalized surgery and close collaboration between surgeon and engineer in cases where advanced techniques were required (3D, three dimensional; MIS, Mimics Innovation Suite; 3D MAC-WRNMMC, 3D Medical Application Center – Walter Reed National Military Medical Center. CT; Computed tomography, MRI; Magnetic Resonance Imaging.)

The printed models of group 1 and 2 were made by the material extrusion or fused deposition modeling (FDM) with acrylonitrile butadiene styrene (ABS) material, using the UP Box (Beijing Tiertime Technology, Beijing, China). The model from group 3 was made from a Binder Jetting Machine, ZPrinter 650 (3D Systems, South Carolina, United States) using VisiJet PXL materials. A step-wise workflow of the 3D printed model process is shown in Fig. 1.

Group I: Anatomical models with major vascular consideration during surgery - proximal tibial exostosis.

**Fig. 2:**
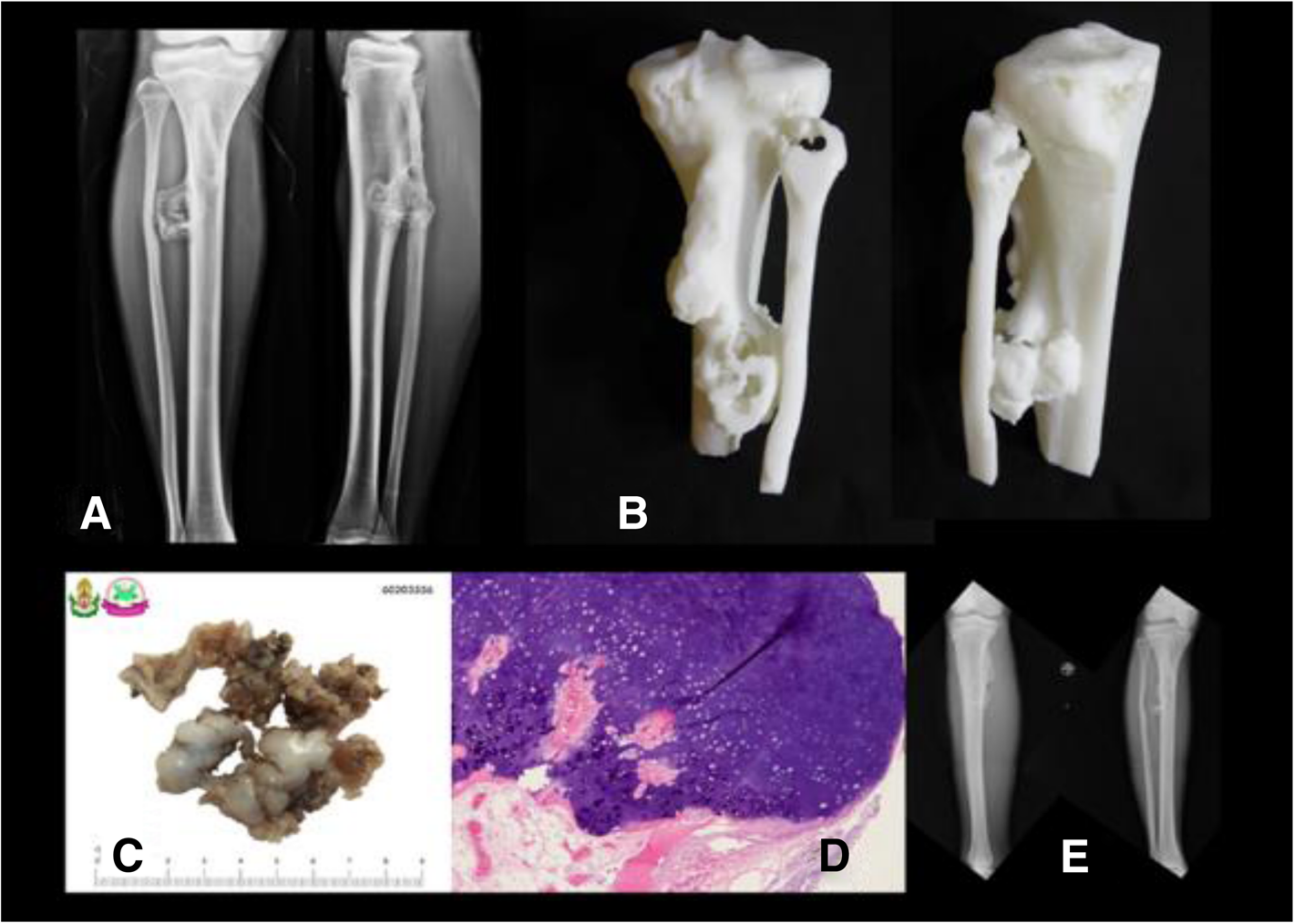
**a** Pre-operative x-ray of proximal tibial exostosis, (**b**) real-size 3D printed model from fused deposition modeling (FDM) printer with acrylonitrile butadiene styrene (ABS) material as a physical simulation for preoperative planning, (**c**) + (**d**) gross- and micropathology of exostosis, (**e**) postoperative x-ray after performing marginal excision

An exostosis on the posterior part of the proximal tibia could be difficult to access due to nearby structures and neurovascular bundles (Fig. 2). This anatomical model could guide the resection plane intra-operatively.

Group I: Anatomical models with major vascular consideration during surgery - distal femoral osteosarcoma.

From the femoral vessels origin, they travel through the femoral triangle and then inferomedially through the thigh within the sartorial canal. In the distal third of the thigh, the femoral vessels exit the sartorial canal by traveling through the adductor hiatus, after which they continue as the popliteal vessels. Thus, the surgical approach for distal femoral resection begins with a long medial incision at the midthigh. It allows simple and safe exploration of the superficial femoral vessels within the sartorial canal and popliteal space. It also permits distal extension of the incision to develop a medial gastrocnemius muscle transposition for allograft or prosthetic coverage [16].

**Fig. 3:**
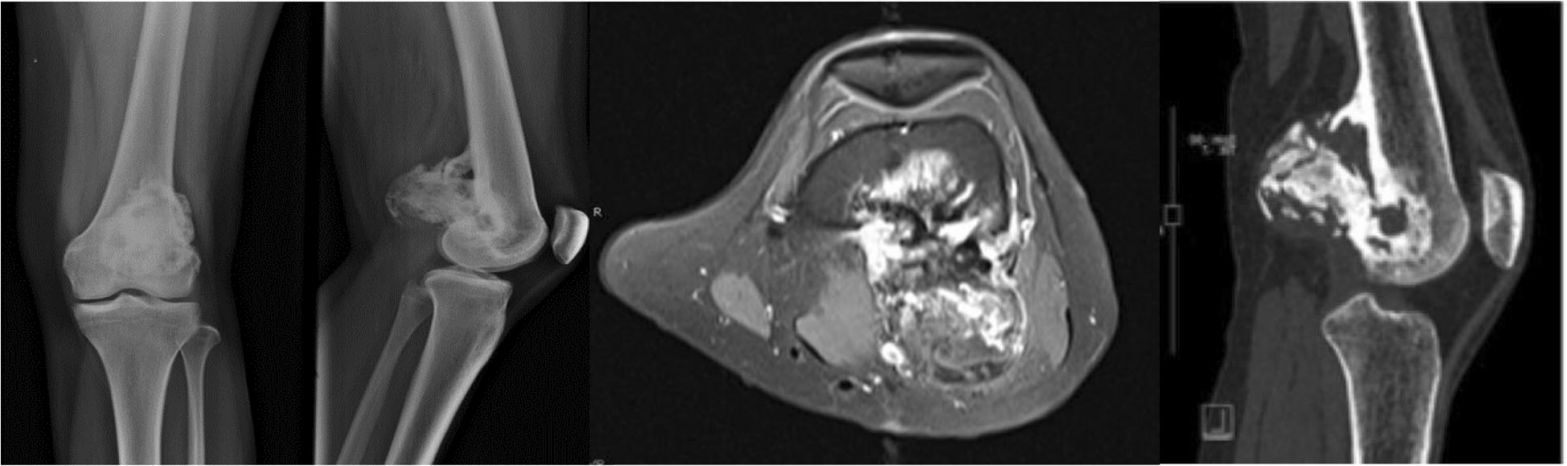
27-year-old female, parosteal osteosarcoma left distal femur (**a**) Plain film images of both knees demonstrating bony mass extension from the posterior part of the distal femur. **b** Axial MRI reveals infiltrative intramedullary lesion and soft tissue component showing heterogenous enhancement with intralesional calcification, (**c**) sagittal CT scan reveals bony mass involving distal femoral metaphysis from the posterior aspect

**Fig. 4:**
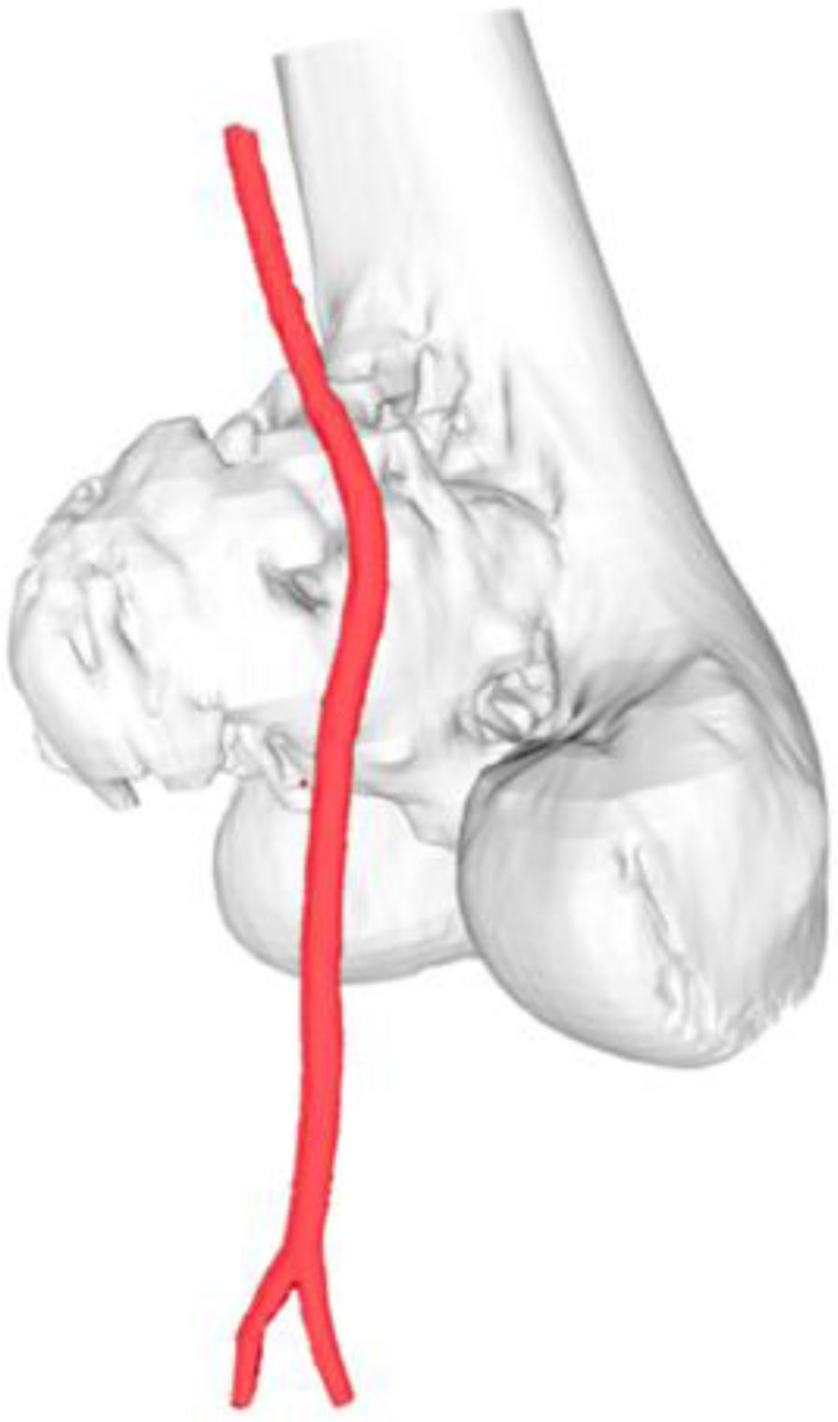
Postprocessing DICOM image to STL file demonstrating 3D rendered model of the relationship between the parosteal osteosarcoma left distal femur and the femoral artery

**Fig. 5:**
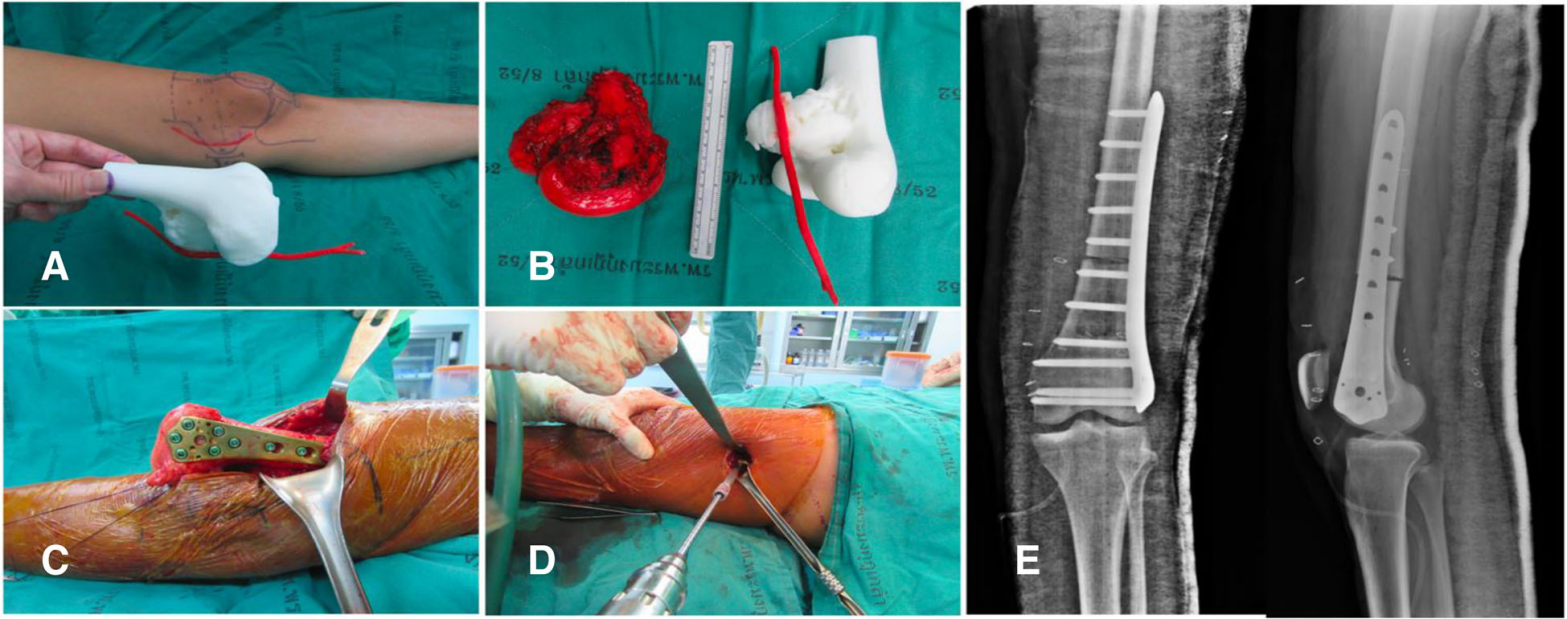
**a** 3D model created from fused deposition modeling (FDM) printer with acrylonitrile butadiene styrene (ABS) material that allowed us to measure the arterial positioning and osteotomy site before operating then applying in the surgical field, (**b**) comparing size and characteristics between gross tumor pathology and 3D model, (**c**) after tumor resection, allograft reconstruction was performed with the distal femoral locking compression plate, (**d**) according to the small incision on the medial side, a mini-open lateral incision must be made to be able to purchase the screws from the lateral side, (**e**) Demonstrating the postoperative film images of the distal femoral allograft reconstruction

Using the 3D model of the distal femoral parosteal osteosarcoma with femoral arterial implication can aid a surgeon to locate the general position and proximity of an artery in relation to the surrounding tissues (Figs. 3 and 4). This provides proof that the operation can be performed by making a smaller incision without using conventional techniques to identify the artery in the sartorial canal. Our surgical team measured the arterial positioning and osteotomy site on the 3D model before operating then applied in the surgical field (Fig. 5). This model verified the arterial position, confirming that the femoral artery was not in the location that we were approaching to resect the distal femur. Hence, we felt identifying the femoral artery intra-operatively was unnecessary because of the printed model. As a result, we assumed the surgical incision length, operative time and patient blood loss would be a reduced.

Group II: Anatomical models with bone defects which were intended to be used for substantial instrumentation - CA thyroid with acetabular metastasis.

**Fig. 6:**
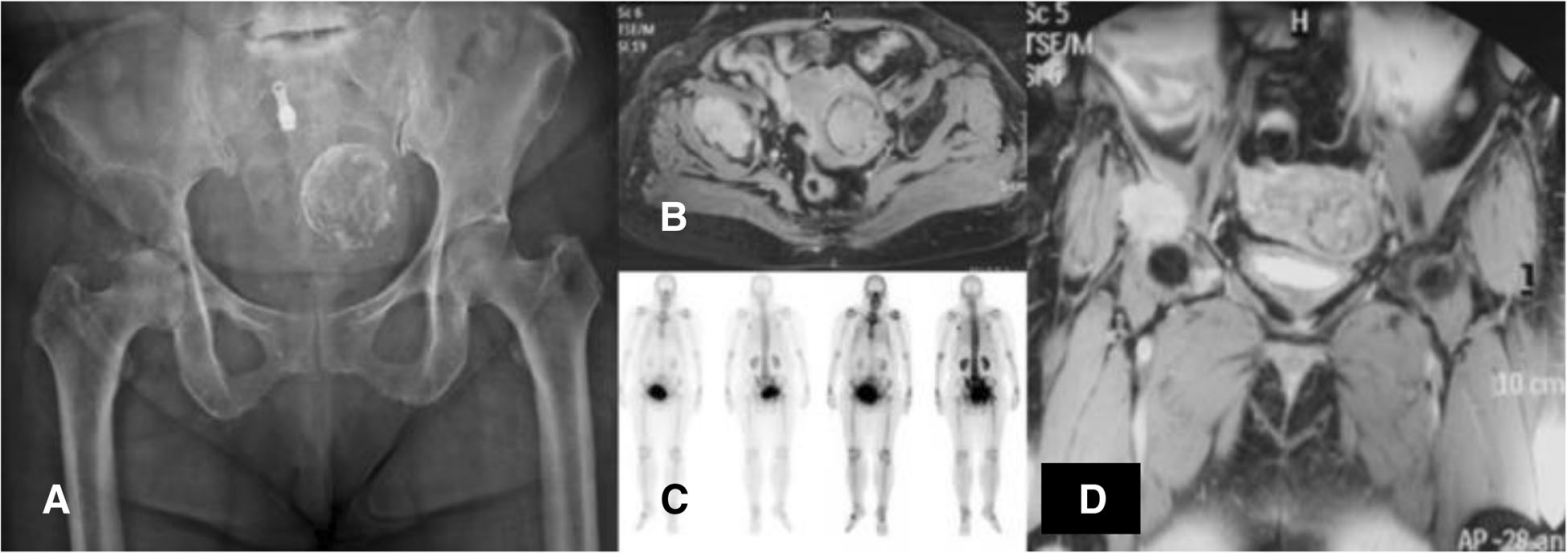
**a** Film images of both hips AP showing osteolytic lesion in the supra-acetabular area, (**b**) axial MRI revealing lesion in the right hip with soft tissue extension, (**c**) bone scan revealing increased uptake on right acetabulum, (**d**) coronal MRI showing lesion on the supra-acetabular area with cortical breakthrough

**Fig. 7:**
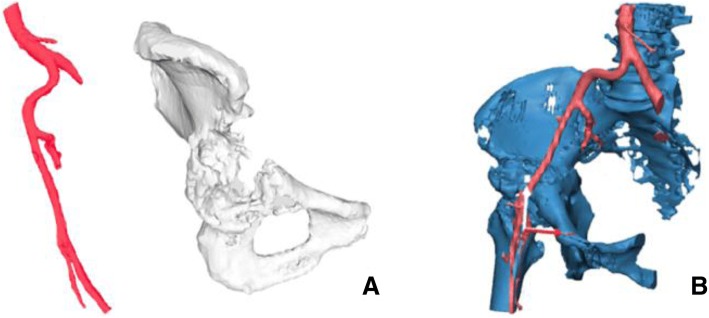
3D model of the right hemipelvis and external iliac artery showing the continuity of the anterosuperior acetabular defect. **a** An isolated part of the vascular and acetabular bone defect in the 3D models. **b** Combining 2 anatomical landmarks (the pelvis and external iliac artery) as the surgical planning outlined

Operations encompassing the pelvic area are delicate due to the approach and their complexity. Controlling bleeding is also especially difficult in the acetabular area. Therefore, surgical procedures for acetabular reconstructions must be handled carefully due to higher risk factors and complications, such as hip dislocation and limb length discrepancy. The use of 3D printed models of the hemipelvis with the external iliac artery could be effective in planning the operation regarding the surgical approach and selection of the acetabular supporting ring. This data provided helpful information to determine the type and size of the prosthesis and its ideal position on the iliac bone. This case involved a 53-year-old woman presenting papillary thyroid carcinoma with lung and right peri-acetabular metastasis, presenting hip pain and in need of a wheelchair to ambulate (Figs. 6, 7).

Group III: 3D printed extracompartmental bone tumor requiring fused CT and MRI images - chondrosarcoma.

**Fig. 8:**
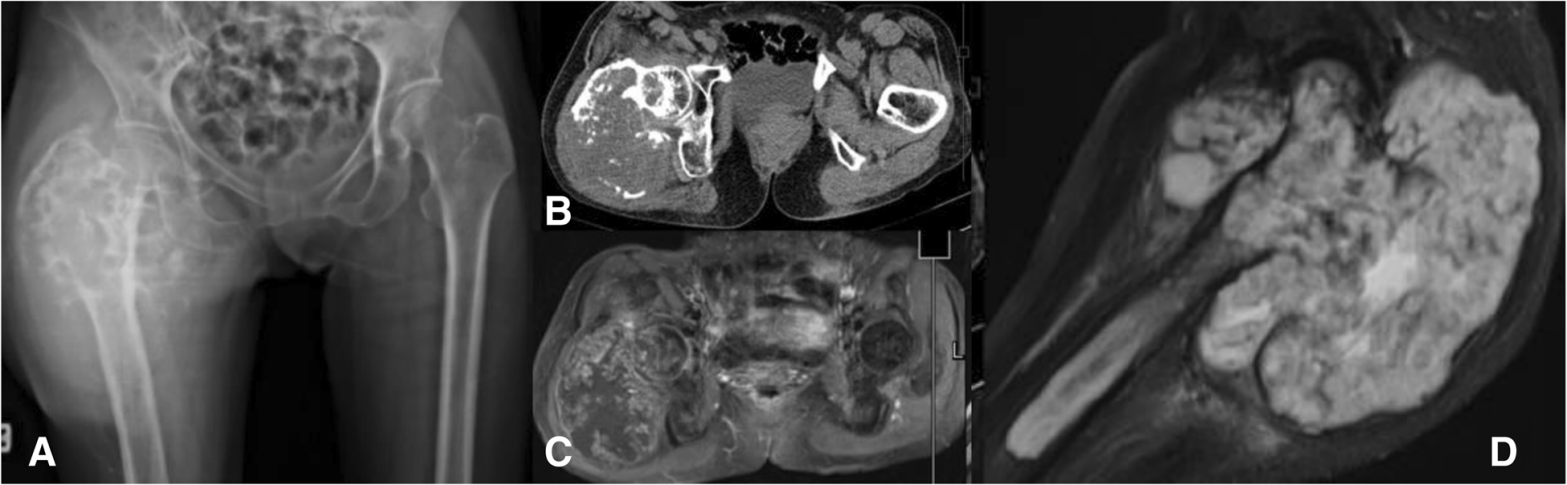
A 73-year-old female, with chondrosarcoma right proximal femur. **a** The film images of both hips showing large bony lesion with suspected chondroid matrix within the right proximal femur, (**b**) Axial CT scan revealing cortical breakthrough with soft tissue extension, (**c**) Axial MRI revealing large mass with heterogeneous enhancing lesion, (**d**) sagittal MRI revealing tumor margin extending into the mid-shaft of the femoral medullary canal

**Fig. 9:**
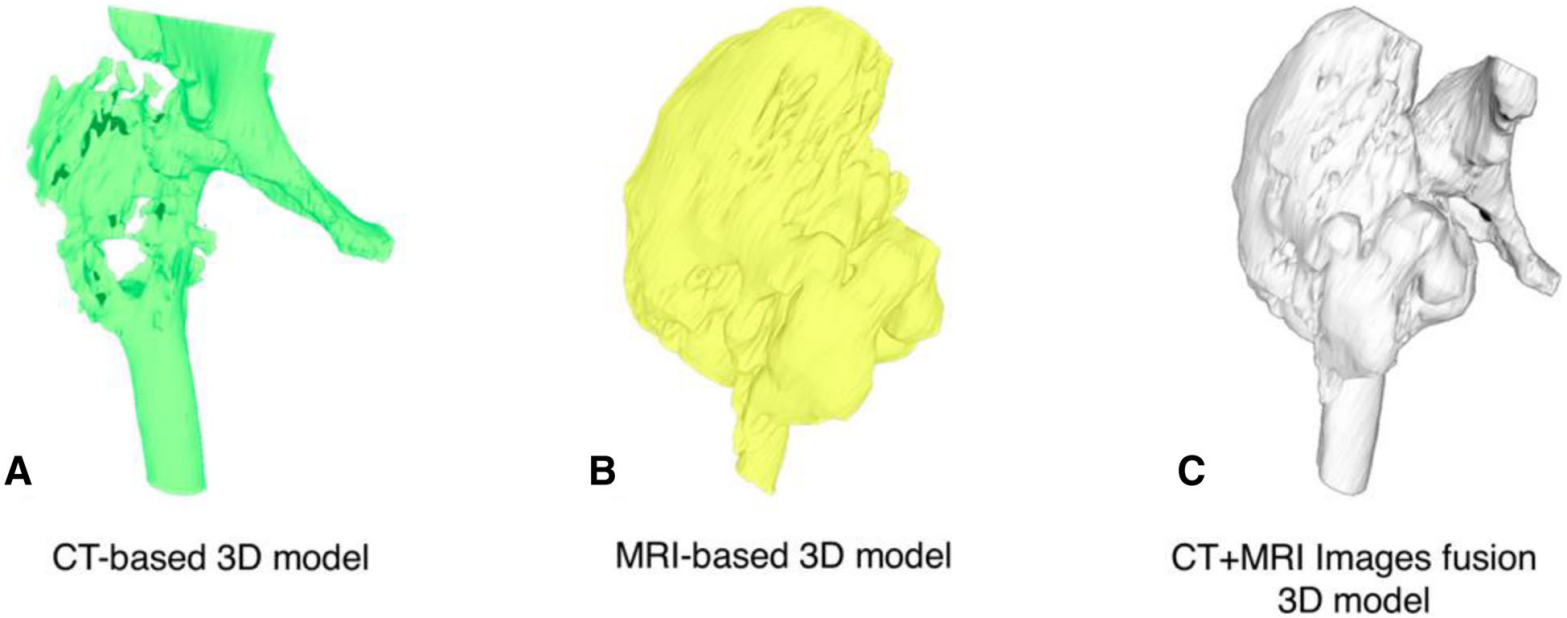
3D proximal femur was segmented by the thresholding process in CAD software in each of the CT-based and MRI-based radiographic files. Outlining the extent of the tumor and its tumor volume created for the 3D bone tumor model base on CT and MRI files as shown in the following Figs. **a**, **b**. CT and MRI images data were fused to construct 3D models of the respective tumor regions as shown in Fig. **c**. CAD; Computer-aided design

The technique for combining the CT scan and MRI is beneficial to evaluate the vital structures and surgical margins before operating because the surgeon can observe the 3D model in 360 degrees and use the model as a physical simulation before the actual surgery. The technique involves fusing CT and MRI images together. This increases the complexity needed to incorporate advanced engineering technology in reconstruction procedures to accurately and reliably depict the surgical margins, if still uncertain. However, there is always some error in registrations. Slice thickness can play a role in this error, and it is at the discretion of the provider if appropriate. For example, in the case of the extracompartmental chondrosarcoma proximal femur (Fig. 8), the 3D model was produced to evaluate surgical margins (Fig. 9). In this case, the surgical team tried to combine initially with a CT scan containing 5 mm thickness combined with MRI images 7 mm slices thick.

## Results and discussion

Group I: Anatomical models with major vascular consideration during surgery - proximal tibial exostosis.

While using anatomical models during surgery, postoperative time was 1 h 30 min and estimated blood loss was minimal. However, the limitation was no similar case could be found to compare between used and unused 3D models.

Group I: Anatomical models with major vascular consideration during surgery - distal femoral osteosarcoma.

**Fig. 10:**
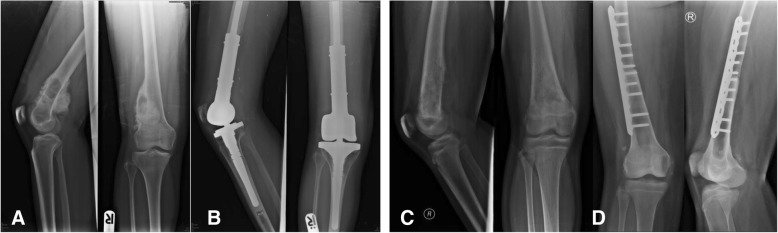
Two comparison distal femoral osteosarcoma cases treated without using 3D model for assistance. **a** The first case showing the x-ray finding of the dedifferentiated parosteal osteosarcoma right distal femur, (**b**) Performed wide resection of 17.5 cm length and reconstructed by rotating hinge knee endoprosthesis, (**c**) The second case showing the x-ray finding of conventional osteosarcoma right distal femur, (**d**) Performed wide resection of 20 cm and reconstructed with the distal femoral allograft and locking compression plate

**Fig. 11:**
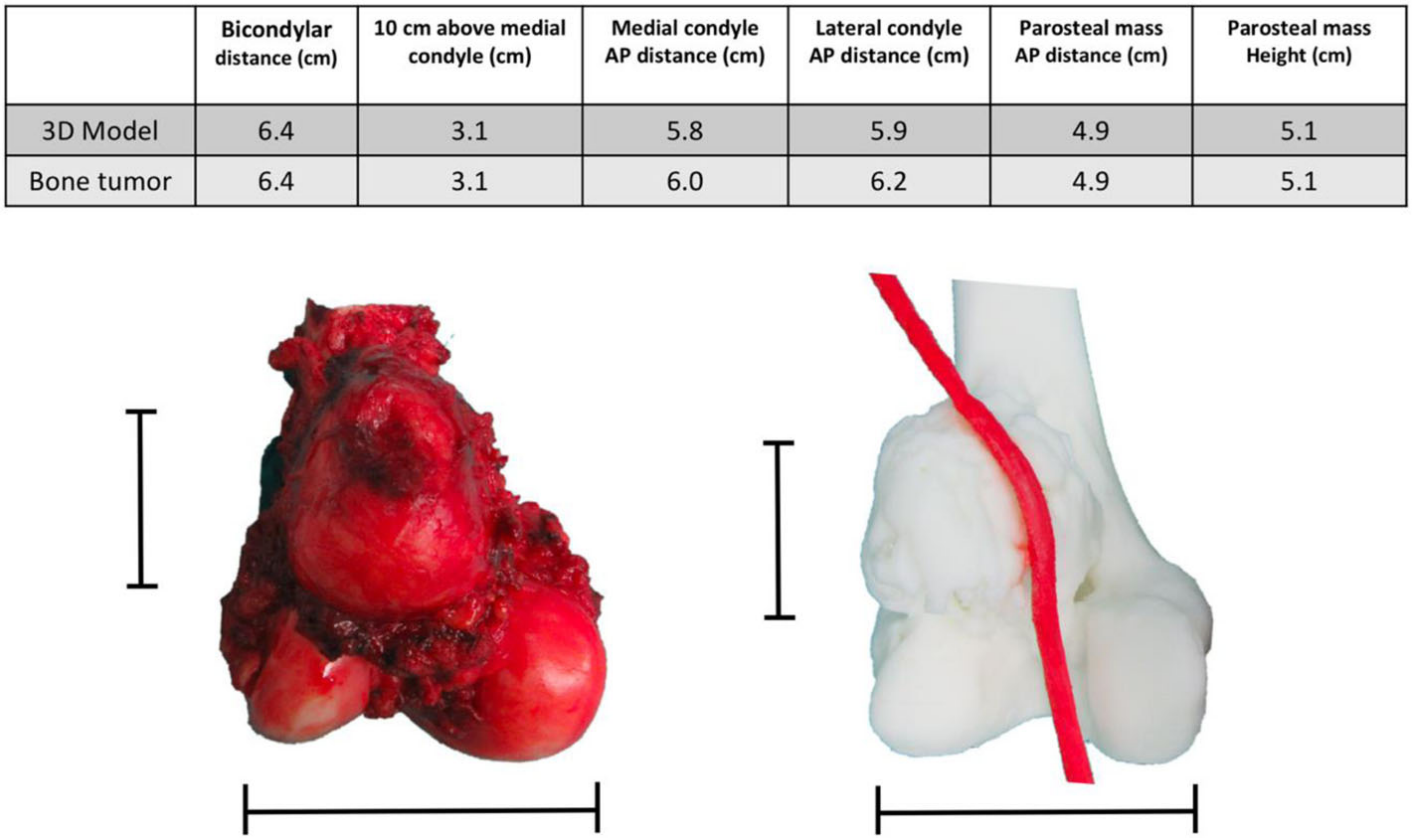
Table and pictures of differences between anatomical size measuring of 3D model and patient’s specimen showing little to no difference in any bony planes excluding cartilage thickness

Postsurgery, the clinical outcome reduced operative time, while estimated patient blood loss and length of the surgical incision was 208 min, 250 ml and 12 cm (medial), 4 cm (lateral), respectively. The locking compression plate was placed on the lateral side of femur, using a lateral incision, and in this case, was performed because the medial incision was too small. We could not access the proximal locking screws, which were on the lateral side from the single medial incision. Comparing with two previous distal femoral osteosarcoma cases with a similar diagnosis, the surgeon was identical and within the same year the result showed a significant reduction (Fig. 10). The statistics for these 2 cases are as follows: durations were 421 and 405 min, blood losses were 1800 and 2000 ml and the incision lengths were 29 and 32 cm (medial), respectively (Table 1). These values were consistent with other reports where the use of 3D models could significantly decrease operative time and blood loss [5]. In addition, the researcher also used the 3D model to compare the pathological distal femoral resection. The results, shown in Fig. 11, imply that the size of the 3D model was consistent with the resected bone of the patient except for the cartilage which could not be measured by CT scan.

**Table 1:** Different outcomes between using 3D models and conventional techniques

*Parosteal osteosarcoma*	3D model assisted	Conventional technique -1st case-	Conventional technique -2nd case-
Operative time (min)	208	421	405
Estimated blood loss (ml)	250	1800	2000
Surgical incision length (cm)	12 (medial)	29 (medial)	32 (medial)
	4 (lateral)		
Bone resection length (cm)	10.5	17.5	20

Group II: Anatomical model with bone defect intended to be used for substantial instrumentation - CA thyroid with acetabular metastasis.

**Fig. 12:**
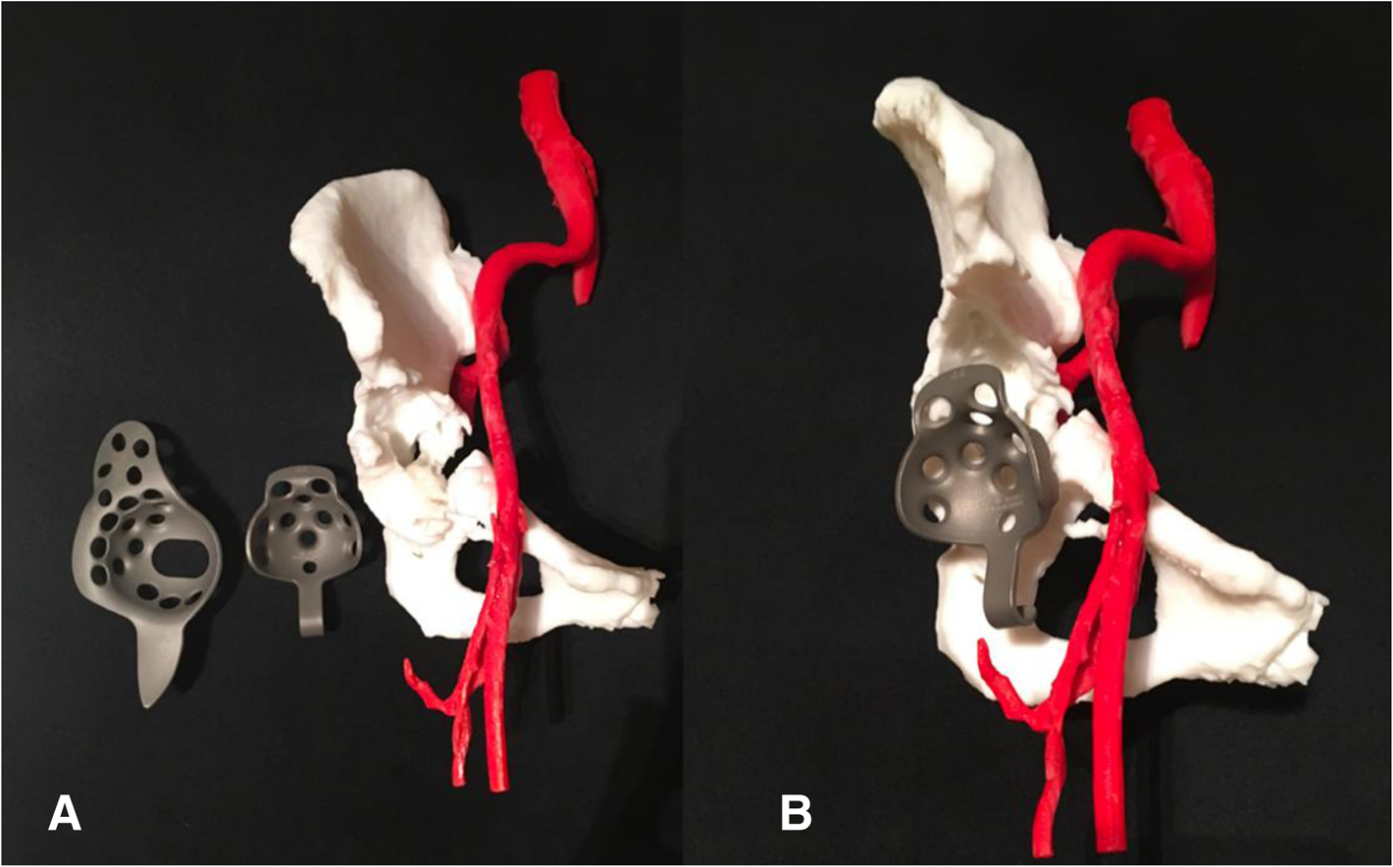
**a** 3D pelvic with external iliac artery model created from fused deposition modeling (FDM) printer with acrylonitrile butadiene styrene (ABS) material showing sizeable peri-acetabular bone defect as a pre-operative physical simulation assisted for implant selection between Burch-Schneider Cage and Ganz ring. **b** Ganz ring, size 44 with three posterior screws selected as pre-operative planning, the rest filling bone defect with PMMA

**Fig. 13:**
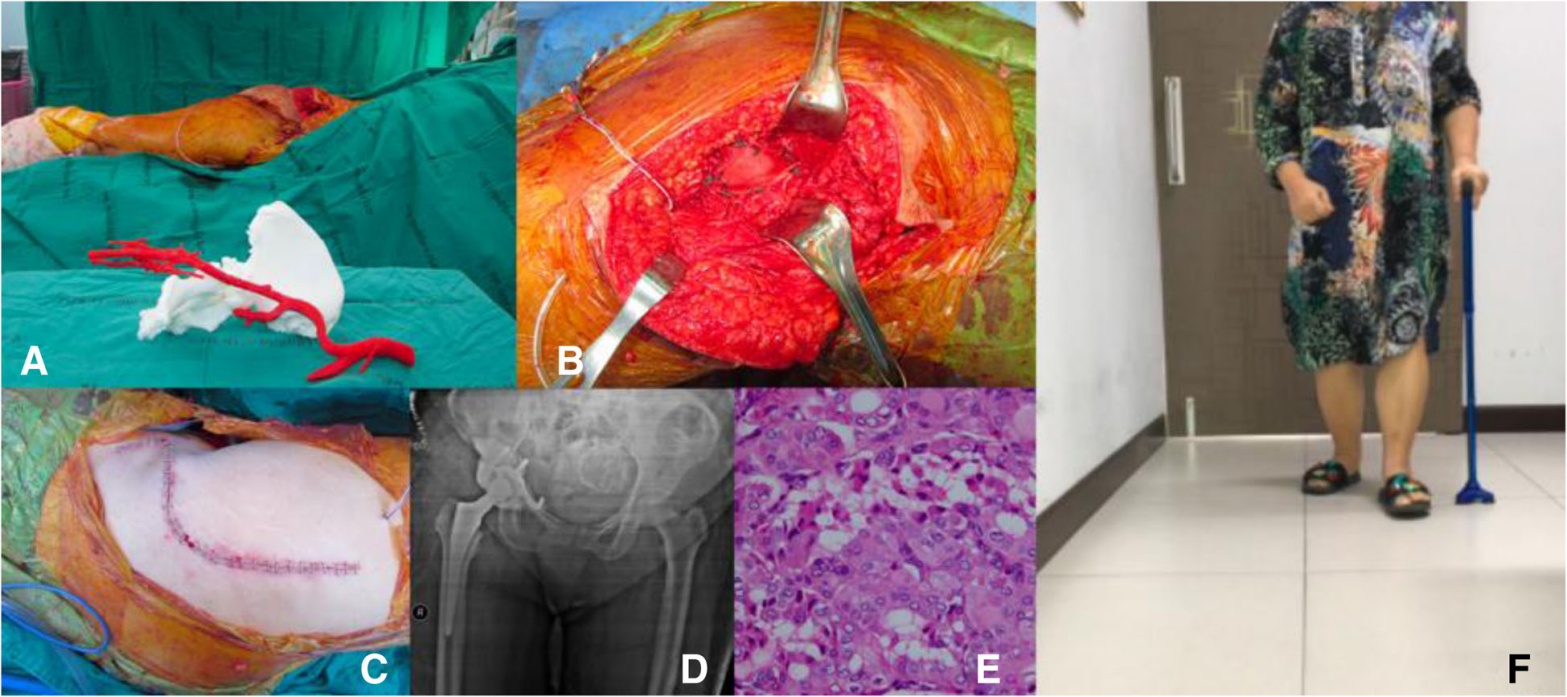
**a** 3D pelvic with vascular printing model in intra-operative simulator assisted surgery. **b** Achieving the surgical plan by removing the tumor and replacing space with cement then reconstructing the acetabular defect with Ganz ring, size 44, with 3 posterior screws surrounded by mesh tube as planned. **c** The surgical approach was closed. **d** Postoperative film showing the Ganz ring position with a cement spacer. **e** Official pathologic report showing metastatic thyroid cancer, papillary type, (**f**) At 2-week follow-up, the patient could walk well with one cane

By using the real-size patient’s 3D hemipelvis model, we were able to measure the diameter of the acetabular cup as 44 mm and the supero-inferior, anteroposterior length and depth of supraacetabular defect as 24, 35 and 24 mm, respectively. From this data, the surgeon could select the most appropriate prosthesis, Gantz ring, size 44 with three screws at the posterior position, pre-operatively (Figs. 12 and 13).

Group III: 3D printed extracompartmental bone tumor requiring fused CT and MRI images - chondrosarcoma.

**Fig. 14:**
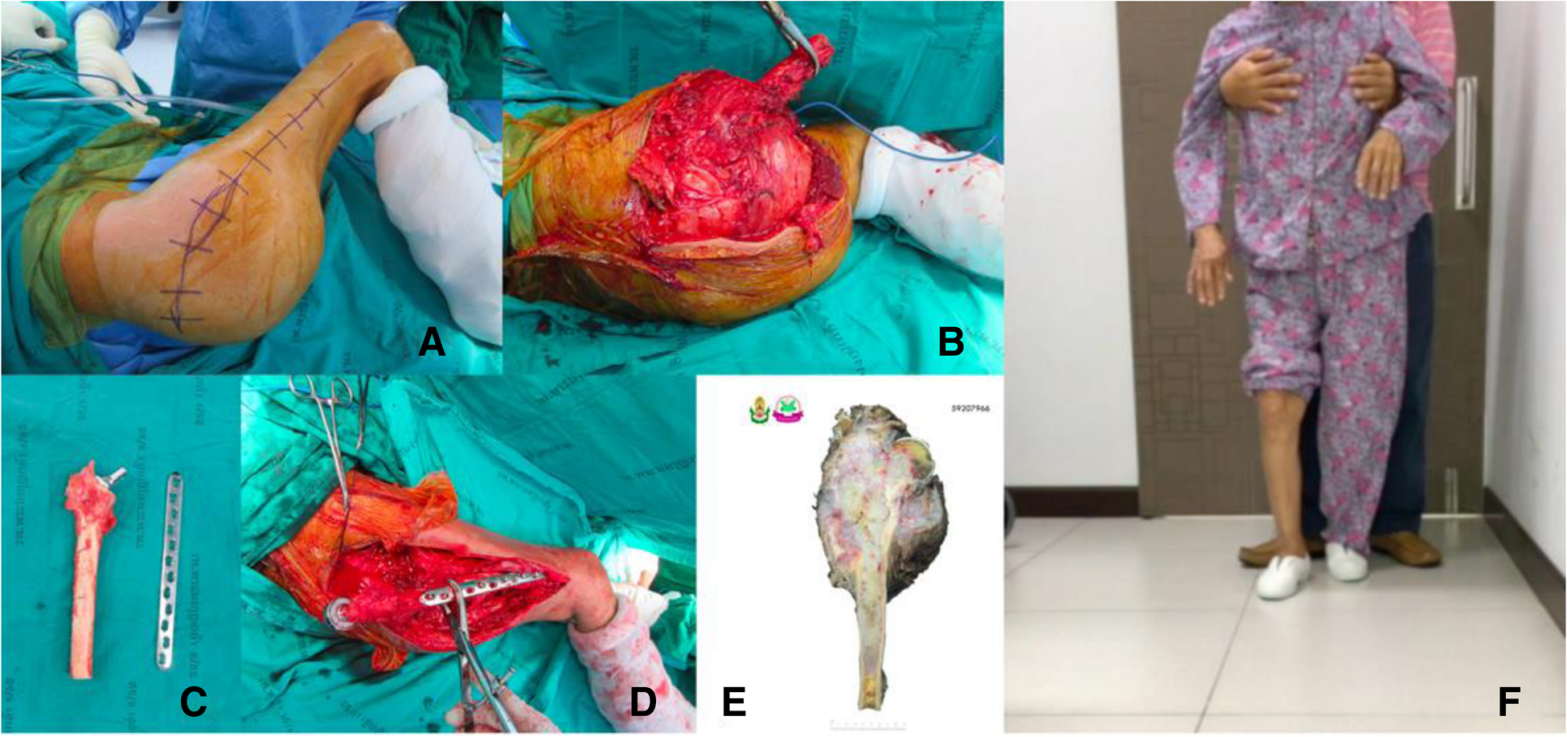
Intra-operative resection was made as planned with the margin-evaluated 3D model. **a** Surgical approach for tumor resection (**b**) Performing the wide resection, showed the gross pathology similar to the 3D model. **c** + **d** Allograft-prosthesis composite selected for reconstruction. **e** Gross pathology showing all margins were free. **f** Ten-month follow up could see the clinical bony union on the junction. Patient can stand and amble with a helper

**Fig. 15:**
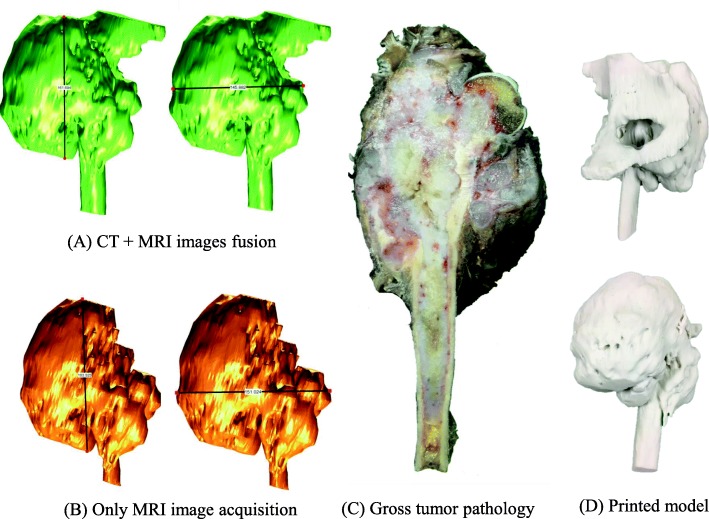
Validating the accuracy between STL file from CT + MRI images acquisition (**a**), STL file from only MRI image acquisition (**b**), gross tumor pathology of chondrosarcoma (**c**), and printed model was made from the Binder Jetting Machine, 3D Systems ZPrinter 650 (**d**). From the resource of 5 mm thickness, CT scan and 7 mm thickness, MRI, the accuracy of this model was shown in Table 2

Postoperative validation of the accuracy was assessed between STL file from MRI alone, STL file from MRI + CT images fusion, Printed model, Gross tumor pathology, and MRI image. The reference points for the longitudinal plane are tip of the uppermost and lowest pole of extra-skeletal part of tumor mass. The reference points for horizontal plane are the edge of medial and lateral sides. In this case, we did not measure the length of tumor extension to intramedullary diaphysis (Figs. 14, 15). The comparing result was shown in Table 2.

**Table 2:** Comparing the geometric measurement outcomes between STL files, printed model, gross pathology, and MRI image

	STL data from MRI	STL data from MRI + CT	Printed model	Gross tumor pathology	MRI image
Longitudinal length (cm)	16.21	16.16	16.2	15.5	15.6
Horizontal length (cm)	15.08	14.52	14.3	12.0	11.6

According to the 7 mm thickness, MRI, we accept the error range 7 mm. Although the longitudinal length is in the acceptable error range, the horizontal length is not. The horizontal length of 3D model is not precise because tumor mass blend with femoral head and acetabulum leading to difficult to distinguish the signal differences between tumor mass, proximal femur, and acetabulum in the post-processing DICOM to STL. When we compare the STL data with printed model from the Binder Jetting Machine, 3D Systems ZPrinter 650, the results are within 0.2 mm.

3D printing technology has gained increasing popularity for practical work, especially in orthopedics, which exhibits the most significant share of 3D printing surgical domains (45.18%) and the second largest share belongs to maxillofacial surgery (24.12%) [17]. The trend of 3D printing technology in orthopedic surgery could be classified in three parts, namely, anatomical models, custom surgical guides and patient-matched implants. In addition to providing three-dimensional visual stimulus, a 3D printed model also provides crucial tactile stimulus. This can be exploited to great advantage using education methods in teaching anatomy, selecting the optimum of operative hardware, improving fracture characterization in complex cases, surgically correcting severe scoliosis, better delineating resection margins, and informing patients more completely when obtaining consent by explaining with tangible 3D models [18, 19].

Custom surgical guidelines or patient-specific instruments (PSI) for intra-operative guidance are frequently used to increase the accuracy of bone cuts during complex resections and navigating drill guides especially in pelvic and spinal surgery. Pelvic resections are difficult procedures due to limited working space, complex geometry and decreased intra-operative visibility. Pedicle screw fixation using the posterior approach is a standard operation for spinal surgery. However, some locations present challenges such as the atlantoaxial vertebra owing to the high risk of blood vessel and nerve damage restricting guideline application [20]. As a result, pelvic resections by PSI and patient-specific 3D printed templates for drill navigational guidance for pedicle screws are especially important and becoming more popular.

Creating patient-matched implants is supported by 3D printing technology in multiple ways including anatomical models used as a baseline for manual design, intra-operative or immediately pre-operative bending/fitting by surgeons, models used as a manufacturing tool/pattern and digital design and 3D printing of implants directly in an implantable material. According to a design software standpoint, there have been a lot of development from primarily designing by hands, even when a designer would do this work digitally, to the tools which can totally automate many of these design tasks, making the labor intensive effortless. In addition to saving time and money on labor, other benefits are reproducibility and standardization. We believe in the next 5 years, the automation will be able to change the economics and accessibility of personalized, patient-matched design [21]. A few examples of patient-matched implants are patient-matched entire prosthesis of the first metacarpal [8], 3D printed acetabular prosthesis [10], low cost customized cranioplasty using a 3D digital printing model [21] and salvage surgery using an ankle fusion titanium cage using direct 3D printing [21]. Furthermore, the future possibility of printing 3D bone repair scaffolds integrated with tissue engineering, i.e., “Bioprinting” would allow implantation within the body that would be generated to fit particular defects [22, 23]. In the advanced research of 3D musculoskeletal printing, custom-built 3D printed scaffolds using polycaprolactone (PCL) seeded with human adipose-derived stem cells (hASCs) have been created. These scaffolds promoted the induction of hASCs to form vasculature and bone. Regarding cartilage regeneration, 3D printed scaffolds, fabricated by biocompatible hydrogels or water-absorbable cross-linked networks, create a cartilaginous matrix in which chondrocytes and stem cells are encapsulated within the alginate hydrogels and can remain viable and metabolically active after implantation. This treatment could potentially promote chondral regeneration after cartilage injury [18].

The quality assurance (QA) processes were established to assess the accuracy and precision of each step during the 3D printing process. There are image acquisition, segmentation and processing, printing process, validation of printed model, and phantom-based QA processes [24]. According to our margin-evaluated model of chondrosarcoma is not quite accurate in the horizontal plane, we can analyze the causes by using the QA approach. In this case, the acquired volumetric image data which are CT scan 5 mm thickness and MRI 7 mm thickness play the critical role as a cause of inaccuracy of this model, and future imaging series used for 3D model reconstruction will be substantially decreased to approximately 1 mm in slice thicknesses. For the segmentation process, we combined the automatic segmentation tools with hand segmentation techniques. For the 3D printing process, we used the Binder Jetting Machine, 3D Systems ZPrinter 650. We validated our printed model by manual techniques in centimeter as we showed in Fig. 15 and Table 2 however we did not use the phantom in this case. Although the detail of bone in an MRI scan is usually less, the option of evaluating bone tumor with soft tissue extension by MRI alone for segmentation is able to do by done using sequences like a Zero Echo Time (ZTE) on a GE scanner or Ultra-short Echo time (UTE) on a Siemens scanner. Therefore it is possible, but not all facilities will have access to these scanners. The newer MRI Scanner feature sequences may allow both bone and soft tissue to be reconstructed from a single image set. This would be beneficial in reduce patient radiation exposure. However, the image fusion is easier and faster to segment the bone from CT than the routine MRI.

The measurement in Table 2 showed not much difference in term of soft tissue extension in vertical plane. However, the pelvic shape of STL file that came from MRI alone is not anatomical shape comparing with CT and MRI (Fig. 15). As a result, CT and MRI images fusion can provide more benefit than MRI alone in term of accuracy in complex bony area such as pelvis.

Currently, a growing number of radiology-based in-hospital 3D printing labs have been established along with imaging experts and engineers. In our opinion, imaging experts are still needed to diagnose the scan and formulate a report. This report can then be used by others to aid in the DICOM segmentation and model production. In addition, if any questions arise by the medical staff/engineer performing the modeling a radiologist should be consulted. The successful implementation of 3D printing services requires the cooperation of surgeons or physicians, radiologists, medical physicists, and engineers [25]. This inter-professional communication is essential to understand the practical potential and limitations of each other’s discipline resulting in reduced time and costs of productions to create anatomical models, modified anatomical models and custom-made surgical guidelines. The skeletal radiographic system differs from internal organs imaging, so orthopedic surgeons could mostly interpret CT and MRI by themselves. In our opinion, orthopedic surgeons, or other medical professionals, can become proficient at imaging an CAD software with the proper training and motivation.

Regarding clinical aspects, bone tumors vary concerning location and characteristics, requiring a more personalized surgical approach. These types of cases may require 3D printed models and devices leading to a more personalized medical approach and greater individualization, thus producing time-consuming procedures. For this research, over 5 months, 4 cases were treated and each needed different technological applications. For three of these cases, the surgeon designed and printed 3D models individually, namely, two models using vascular printing (the pelvis and distal femur) and one 3D-model of the exostosis posterior part of the proximal tibia. For the final case, the surgeon cooperated with the engineer to fuse CT and MRI images of the proximal femur. Although our 3D printed model shows an impact on operative time, blood loss and incision length in case of distal femoral osteosarcoma, group 1, the comparison cases are different in term of bone resection length and methods of reconstruction. These are the limitations for this comparison.

Orthopedic oncology is a branch of education with substantial interests in 3D printing because bone tumors cases have many characteristics, requiring different treatment decisions and personalized surgery. Hence with this technology, the patient can receive treatment that is optimally suited to their physicality. Personalized surgery has become a growing topic of interest. In our opinion, the key to the further widespread adoption of patient-matched 3D printed technology is proof that this option provides better patient care and a risk, time, and cost reduction in the surgical procedure and rehabilitation. This would then benefit the hospital, insurance, and personal payer. Further software automation and better direct implant manufacturing using 3D printing could push prices down, so many patients could be able to adopt patient-matched implants in many different areas of the body.

## Conclusion

3D printing technology will play an important role in orthopedic oncology. It can provide printed models, custom surgical guides, and patient-matched implants. The best learning objective for surgeons would be to understand the basic operations of 3D printing and its technical limitations. Vice-versa, it would be vital for engineers to understand the basic concepts of anatomy and surgical approaches. This understanding could be key to the communication efficiency between surgeons and engineers in designing medical materials using 3D printing technology.
